# Effect of Serum Interleukin-6 Concentration on the Prognosis After Radiotherapy for Patients With Hepatocellular Carcinoma

**DOI:** 10.1155/cjgh/4696097

**Published:** 2024-11-21

**Authors:** Yong Hu, Yongkang Zhou, Shisuo Du, Wenchao Zhu, Yixing Chen, Zhaochong Zeng

**Affiliations:** Department of Radiation Oncology, Zhongshan Hospital, Fudan University, 180 Feng Lin Road, Shanghai 200032, China

**Keywords:** hepatocellular carcinoma, interleukin-6, prognosis, radiotherapy, serum inflammatory factor

## Abstract

**Objective:** The aim of this study was to explore the association between interleukin-6 (IL-6) concentration before radiotherapy (RT) and the prognosis after RT for patients with hepatocellular carcinoma (HCC).

**Methods:** The clinical data for 101 patients with HCC who received RT from October 2016 to June 2021 were retrospectively analyzed. In these patients, the tumors were confined to the liver, and IL-6 concentration was measured before RT. The survival rate was calculated using the Kaplan–Meier method, and the Cox proportional hazards regression model was used to explore the independent factors affecting the patients' prognosis. *X*-tile software was used to obtain the optimal cut-off value of pre-RT IL-6 concentration (7.8 pg/mL) for overall survival (OS).

**Results:** The 1-, 2-, and 3-year OS rates were 84.4%, 55.8%, and 34.7%, respectively, for patients with a pre-RT IL-6 concentration > 7.8 pg/mL versus 96.0%, 80.1%, and 80.1%, respectively, for those with a pre-RT IL-6 concentration ≤ 7.8 pg/mL. The OS rates of the two groups were significantly different (*p* < 0.001). The median progression-free survival (PFS) time was 7.5 months versus 15.1 months for patients with pre-RT IL-6 concentrations > 7.8 pg/mL and ≤ 7.8 pg/mL, respectively (*p*=0.001). Pre-RT IL-6 concentration was an independent prognostic factor of OS (hazard ratio [HR] = 3.421, 95% confidence interval [CI]: 1.477–7.927, *p*=0.004). Pre-RT IL-6 concentration (HR = 2.235, 95% CI: 1.176–4.246, *p*=0.014) and age (HR = 0.615, 95% CI: 0.383–0.987, *p*=0.044) were independent prognostic factors for PFS.

**Conclusions:** The prognosis of HCC patients receiving RT was worse for those with a pre-RT serum IL-6 concentration > 7.8 pg/mL than those with a pre-RT serum IL-6 concentration ≤ 7.8 pg/mL. Pre-RT IL-6 concentrations may affect the prognosis of HCC patients.

## 1. Introduction

Primary liver cancer is one of the most common and fatal malignant tumors of the digestive system in humans, with the second and fourth highest incidence and mortality rates, respectively, among malignant tumors in China [1–3]. Due to the background of chronic hepatitis B virus infection, China has become a high-incidence area for hepatocellular carcinoma (HCC), which seriously threatens public health [4]. The development of precise radiotherapy (RT) technology, particularly image-guided technology, has greatly improved the prognosis of patients with HCC undergoing RT [5]. Stereotactic body RT (SBRT) is an effective alternative to radiofrequency ablation for small, unresectable HCC [6].

Some inflammatory factors in the tumor microenvironment, such as interleukin-6 (IL-6), play important roles in the occurrence, progression, invasion, and metastasis of tumors [7]. IL-6 promotes the formation of tumor blood vessels, accelerates tumor proliferation, and increases the ability of tumor cells to invade and metastasize [8]. Thus, it is an important factor contributing to resistance to antitumor therapy [8]. Liver tissue is rich in inflammatory immune cells in HCC patients. Inflammatory factors induced by the hepatitis virus cause damage to liver cells, leading to the occurrence of liver cancer. Therefore, it is particularly important to study the effects of inflammatory factors on the efficacy of RT in patients with HCC.

As previously reported, serum inflammatory factors in patients with HCC receiving conventional RT predict the effects of RT, especially the progression in the radiation field [9]. Wei et al. reported the effect of preoperative RT for HCC with tumor thrombus and found that an increase in the baseline serum IL-6 concentration also led to a poor effect of RT [10]. In their study, the patients had advanced disease and the radiation dose was relatively low due to preoperative neoadjuvant RT. However, the relationship between precise radical RT and IL-6 concentration in confined HCC without a tumor thrombus remains unknown, as there are few reports on this topic. In this study, we aimed to determine the predictive value of pre-RT IL-6 concentration on the prognosis after RT of patients with confined HCC without tumor thrombus.

## 2. Materials and Methods

### 2.1. General Information

From October 2016 to June 2021, 101 patients with confined HCC without tumor thrombus, who received RT at Zhongshan Hospital, Fudan University, were enrolled based on the inclusion and exclusion criteria for this study, and a retrospective analysis was conducted. The study protocol was reviewed and approved by the Ethics Committee of Zhongshan Hospital, Fudan University (No. B2021-386R). Informed consent was obtained from all patients.

Patients were included if they (1) had HCC confirmed by pathology or met the clinical diagnostic criteria [4]; (2) had a confined tumor in the liver (without tumor thrombus) and no extrahepatic metastasis, based on imaging examinations, and had received RT; (3) had their serum IL-6 concentration tested before RT; (4) had an estimated lifetime of more than 6 months; and (5) were aged 18–85 years.

Patients were excluded if they (1) did not complete RT as planned; (2) had diffuse HCC in the liver; (3) had an uncontrollable infection; (4) had a malignant tumor in another organ; (5) had a serious heart, lung, or kidney disease; (6) had a serious nervous system disease; or (7) were unable to respond to treatment clearly.

The median follow-up time was 22.0 months, ranging from 6.3 to 64.7 months. Of the 101 patients included in the study, 85 were male and 16 were female. The median age of the patients was 63 years (range: 28–83 years). The maximum diameter of the intrahepatic tumors ranged from 0.8 to 13.3 cm, and the cumulative maximum diameter of the intrahepatic tumors ranged from 0.8 to 13.3 cm. The patients' characteristics are described in Table 1.

### 2.2. Measures of Serum IL-6 Concentration

The serum samples collected from patients on an empty stomach were tested using the IMMULITE 1000 fully automatic chemiluminescence instrument from Siemens, Germany. The diagnostic reagents and supporting calibration materials were all prepared using Siemens's matching reagents, and the measurement method was chemiluminescence.

### 2.3. RT Procedure

RT was performed using helical tomotherapy or an image-guided linear accelerator. The patients were placed in the supine position and immobilized with different devices based on the different treatment machines. Abdominal compression was performed to reduce the breath amplitude in patients who received helical tomotherapy treatment [11, 12]. Free shallow breathing after training was used to manage respiratory movement for patients who received static intensity–modulated RT (IMRT) or volume rotation intensity–modulated RT (VMAT) using an image-guided linear accelerator. Magnetic resonance or positron emission tomography/computed tomography images were used for image fusion to improve the accuracy of gross tumor volume (GTV) delineation when necessary. The GTV was contoured based on visible lesions. The clinical target volume (CTV) was created using different methods, based on the different technologies used; for example, the GTV was expanded by 5 mm to create the CTV for hypofractionated RT [13] but the CTV was exempted for SBRT. The internal target volume (ITV) was determined based on the respiratory movement amplitude of the intrahepatic tumors. A margin of 3–5 mm to the ITV was added to create the planning target volume (PTV) [5, 13]. Image-guided technology was used daily for patients undergoing helical tomotherapy. For patients who underwent IMRT or VMAT, image-guided technology was used before the first and second treatments, after which, it was used weekly.

### 2.4. Follow-Up

After treatment, the patients were followed up every 3 months, and they routinely underwent hematological and imaging examinations, such as routine blood tests, liver and kidney function tests, coagulation function tests, tumor marker measurements, inflammatory factor measurements, chest computed tomography, and abdominal enhanced magnetic resonance.

The modified response evaluation criteria in solid tumors were used to evaluate efficacy based on the following categories: complete remission (CR), partial response (PR), stable disease (SD), and progressive disease. The best response observed in the radiation treatment field was used to evaluate radiation treatment efficacy.

### 2.5. Statistical Analysis

*X*-tile software was used to determine the optimal cut-off value of the pre-RT IL-6 concentration (7.8 pg/mL) for overall survival (OS). SPSS 20.0 (IBM, Armonk, New York, USA) and Prism 8.3.0 (GraphPad, San Diego, California, USA) were used for data processing. Survival rates were evaluated using Kaplan–Meier analysis. The Cox proportional hazards regression model was used to explore independent factors affecting the patients' prognosis. The variables of the Cox proportional hazards regression model *p* < 0.10 in the single-factor analysis were included in the multifactor analysis. Statistical significance was set at *p* < 0.05.

## 3. Results

### 3.1. Overall Treatment Effect

The best response observed in the radiation field was used to evaluate the field response to RT. The objective response rate (ORR) in the radiation field was 94.1%, and 78.2%, 15.8%, and 5.9% of the patients showed CR, PR, and SD, respectively. The median OS rate was not reached, and the 1-, 2-, and 3-year OS rates were 93.7%, 75.2%, and 69.8%, respectively. The median progression-free survival (PFS) time was 12.87 months, and the PFS rates at 1, 2, and 3 years were 51.3%, 33.2%, and 18.9%, respectively. The local control rates in the radiation fields for 1, 2, and 3 years were 91.1%, 89.6%, and 84.4%, respectively.

### 3.2. Relationship Between Pre-RT IL-6 Concentration and RT Treatment Response

There were no significant differences in best response ((*p*=0.723) (Table 2) or ORR ((*p*=0.742) between the groups with pre-RT IL-6 concentrations > 7.8 pg/mL and ≤ 7.8 pg/mL.

### 3.3. Relationship Between Pre-RT IL-6 Concentration and Survival

**Figure 1:**
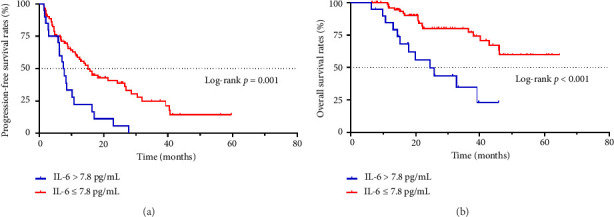
Kaplan–Meier analysis of the relationship between preradiotherapy interleukin-6 (IL-6) concentration and survival.

The median PFS time of patients with an IL-6 concentration > 7.8 pg/mL was 7.50 months, and the PFS rates at 1, 2, and 3 years were 22.2%, 5.6%, and 0%, respectively. The median PFS time of patients with an IL-6 concentration ≤ 7.8 pg/mL was 15.10 months, and the PFS rates at 1, 2, and 3 years were 58.6%, 40.9%, and 24.9%, respectively. There was a statistically significant difference between the two groups (*p*=0.001, Figure 1(a)).

The median OS time of patients with an IL-6 concentration > 7.8 pg/mL was 24.4 months, and the OS rates at 1, 2, and 3 years were 84.4%, 55.8%, and 34.7%, respectively. The median OS time of patients with an IL-6 concentration ≤ 7.8 pg/mL was not reached, and the OS rates at 1, 2, and 3 years were 96.0%, 80.1%, and 80.1%, respectively. The OS time was significantly different between the two groups (*p* < 0.001, Figure 1(b)).

### 3.4. Univariate and Multivariate Cox Regression Analyses

As shown in Table 3, the BED_10_ and pre-RT IL-6 concentrations were statistically significant in the univariate analysis. These two factors were included in the multivariate COX proportional risk regression model analysis. In the Cox proportional hazards model, pre-RT IL-6 concentration was shown to be an independent prognostic factor for OS, with a hazard ratio (HR) of 3.421 and a 95% confidence interval (CI) of 1.477–7.927 ((*p*=0.004, Table 3). The OS time was significantly greater for patients with a pre-RT IL-6 concentration ≤ 7.8 pg/mL than for those with a pre-RT IL-6 concentration > 7.8 pg/mL.

As shown in Table 4, four factors (*p* < 0.10) in the univariate analysis were included in the multivariate COX proportional hazards regression model. The results showed that pre-RT IL-6 concentration (HR = 2.235, 95% CI: 1.176–4.246, *p*=0.014) and age (HR = 0.615, 95% CI: 0.383–0.987, *p*=0.044) were independent prognostic factors for PFS. The PFS time was longer for patients with a pre-RT IL-6 concentration ≤ 7.8 pg/mL than for those with a pre-RT IL-6 concentration > 7.8 pg/mL. The PFS time was longer for patients older than 60 years than those younger than 60 years.

## 4. Discussion

HCC is considered to be an inflammation-related disease and the effects of inflammation range from chronic liver injury to the occurrence and development of HCC. Inflammatory factors, particularly IL-6, play important roles in the occurrence and development of HCC [14]. IL-6 binds to the IL-6 receptor; activates the receptor-related Janus kinase; stimulates phosphorylation; activates signal transducer and activator of transcription 3 (STAT3) to activate downstream signals; exerts antiapoptotic effects; and promotes angiogenesis, proliferation, invasion, and metastasis [15, 16].

Some previous studies have demonstrated that IL-6 may be used as a marker for the auxiliary diagnosis of HCC [17, 18]. The upper limit of the reference value for serum IL-6 concentrations is 3.4 pg/mL in our institution. Among the 101 patients with HCC included in this study, 69 patients (68.3%) had their serum IL-6 concentrations determined (range: 3.4–60.2 pg/mL) before RT and the values exceeded the upper limit of the reference value, and the percentage is similar to AFP [19]. Many previous studies have demonstrated that high IL-6 concentrations indicate poor treatment prognosis for patients with HCC [20–22]. Myojin et al. reported that the circulating IL-6 concentration is a prognostic biomarker for patients with advanced HCC undergoing combined immunotherapy [23]. Wu et al. reported that postintervention IL-6 concentration, rather than the pretreatment concentration or dynamic changes in IL-6 concentration, is a powerful predictor of the tumor response in HCC patients treated with transarterial chemoembolization [24].

The relationship between RT and IL-6 concentration has been the focus of research in the field of radiation immunity. The circulating IL-6 concentration is a predictor of radiation pneumonitis [25, 26]. Matsuoka et al. reported that the blockade of IL-6 signaling, combined with conventional RT, augments the treatment response and survival rate in patients with radioresistant oral squamous cell carcinoma [27]. In this study, we found that HCC patients with a high pre-RT IL-6 concentration were prone to tumor progression, with a reduced OS time, which suggested that IL-6 may weaken the effects of RT.

Increased IL-6 concentrations may lead to a decrease in RT sensitivity [28]. The mechanism for this phenomenon may be related to the fact that inflammatory factors, such as IL-6, promote the repair of DNA breaks after RT and reduce tumor cell apoptosis [29, 30]. DNA damage and repair are determinants of tumor cell sensitivity to radiation. High IL-6 concentrations promote tumor progression after treatment and may decrease RT sensitivity. RT induces the immunogenic death of tumor cells and the generation of injury-related molecules to promote the maturation of dendritic cells, sensitize and activate T cells through MHC-I, and activate the immune system to release a large number of inflammatory factors [31]. RT with concurrent IL-6 inhibition may be a potential therapeutic strategy to increase the radiation response in patients with HCC [32, 33]. In the clinical setting, for such patients, RT specialists also need to actively explore other interventions to improve the prognosis of patients, such as combination immunotherapy, the optimization and adjustment of the RT parameters, and proton or heavy ion therapy. IL-6/STAT3 is an important therapeutic target for HCC [15], and targeted drugs for HCC patients with elevated pre-RT IL-6 who experience relapse may become a promising treatment strategy. In addition, this study also found that patient's age was an independent factor affecting PFS, and patients aged ≤ 60 years were more likely to develop tumors after RT than those aged > 60 years, which may be related to differences in metabolism in these patients.

This study has two main limitations. First, this was a single-center retrospective study and not a large-sample study. However, many patients in this study had participated in a prospective study. Second, this study only analyzed the relationship between pre-RT IL-6 concentration and the prognosis of HCC patients. The post-RT IL-6 concentration and dynamic changes (pre- and post-RT) in IL-6 concentrations were not assessed. It is important to further study the relationship between the dynamic changes in IL-6 concentration and the prognosis of patients with HCC who receive RT and to further explore the mechanism.

## 5. Conclusions

The prognosis of HCC patients receiving RT was worse for those with a pre-RT serum IL-6 concentration > 7.8 pg/mL than those with a pre-RT serum IL-6 concentration ≤ 7.8 pg/mL. Thus, the pre-RT IL-6 concentration may affect the prognosis of HCC patients.

## Figures and Tables

**Table 1:** General characteristics of the patients with HCC.

General characteristics	*n* (%)
Gender	
Male	85 (84.2%)
Female	16 (15.8%)
Age (years)	
Median (range)	63 (28–83)
≤ 60	42 (41.6%)
> 60	59 (58.4%)
History of prior treatment	
Yes	86 (85.1%)
No	15 (14.9%)
HBV/HCV history	
Yes	84 (83.2%)
No	17 (16.8%)
AFP	
Positive	47 (46.5%)
Negative	54 (53.5%)
Number of intrahepatic tumors	
Single	82 (81.2%)
Multiple	19 (18.8%)
Cumulative maximum diameter	
≤ 5 cm	75 (74.3%)
> 5 cm	26 (25.7%)
Child–Pugh class	
A	97 (96.0%)
B	4 (4.0%)
BED_10_	
≤ 80 Gy	31 (30.7%)
> 80 Gy	70 (69.3%)
Pre-RT IL-6 concentration	
≤ 7.8 pg/mL	81 (80.2%)
> 7.8 pg/mL	20 (19.8%)

*Note:* An AFP concentration ≥ 20.0 ng/mL was considered positive and < 20.0 ng/mL was considered negative. Cumulative maximum diameter refers to the cumulative maximum diameter of the intrahepatic tumor. The biological effect dose (BED) was calculated based on the *α*/*β* value of hepatocellular carcinoma (10 Gy, BED_10_).

Abbreviations: AFP, alpha-fetoprotein; HBV, hepatitis B virus; HCV, hepatitis C virus; IL-6, interleukin-6; RT, radiotherapy.

**Table 2:** The relationship between pre-RT IL-6 concentration and the best response of the tumor in the radiation field.

Pre-RT IL-6 concentration (pg/mL)	Best response of the tumor in the radiation field			*p* value
	CR	PR	SD	
> 7.8	15 (75.0%)	3 (15.0%)	2 (10.0%)	0.723
≤ 7.8	64 (79.0%)	13 (16.0%)	4 (4.9%)	

Abbreviations: CR, complete remission; IL-6, interleukin-6; PR, partial response; RT, radiotherapy; SD, stable disease.

**Table 3:** Univariate and multivariate Cox regression analyses of overall survival.

	Univariate		Multivariate	
	HR (95% CI)	*p* value	HR (95% CI)	*p* value
Gender (male vs. female)	1.960 (0.594–6.471)	0.269		
Age (years) (> 60 vs. ≤ 60)	1.026 (0.499–2.109)	0.944		
History of prior treatment (yes vs. no)	0.991 (0.377–2.606)	0.985		
HBV/HCV history (yes vs. no)	1.312 (0.457–3.762)	0.614		
AFP (positive vs. negative)	1.786 (0.859–3.712)	0.120		
Number of intrahepatic tumors (multiple vs. single)	1.492 (0.660–3.370)	0.336		
Cumulative maximum diameter (> 5 cm vs. ≤ 5 cm)	1.675 (0.805–3.487)	0.168		
Child–Pugh class (B vs. A)	3.310 (0.774–14.153)	0.106		
BED_10_ (> 80 Gy vs. ≤ 80 Gy)	0.449 (0.218–0.925)	0.030	0.684 (0.305–1.531)	0.355
Pre-RT IL-6 (> 7.8 pg/mL vs. ≤ 7.8 pg/mL)	4.064 (1.901–8.691)	< 0.001	3.421 (1.477–7.927)	0.004

*Note:* An AFP concentration ≥ 20.0 ng/mL was considered positive and < 20.0 ng/mL was considered negative.

Abbreviations: AFP, alpha-fetoprotein; BED, biological effect dose; CI, confidence interval; HBV, hepatitis B virus; HCV, hepatitis C virus; HR, hazard ratio; IL-6, interleukin-6; RT, radiotherapy.

**Table 4:** Univariate and multivariate Cox regression analyses of progression-free survival.

	Univariate		Multivariate	
	HR (95% CI)	*p* value	HR (95% CI)	*p* value
Gender (male vs. female)	1.372 (0.700–2.686)	0.357		
Age (years) (> 60 vs. ≤ 60)	0.658 (0.413–1.049)	0.078	0.615 (0.383–0.987)	0.044
History of prior treatment (yes vs. no)	1.670 (0.844–3.303)	0.140		
HBV/HCV history (yes vs. no)	1.023 (0.547–1.911)	0.944		
AFP (positive vs. negative)	1.054 (0.658–1.689)	0.826		
Number of intrahepatic tumors (multiple vs. single)	1.465 (0.821–2.616)	0.196		
Cumulative maximum diameter (> 5 cm vs. ≤ 5 cm)	1.538 (0.925–2.558)	0.097	0.771 (0.394–1.506)	0.446
Child–Pugh class (B vs. A)	1.146 (0.359–3.661)	0.818		
BED_10_ (> 80 Gy vs. ≤ 80 Gy)	0.495 (0.301–0.814)	0.006	0.576 (0.296–1.122)	0.105
Pre-RT IL-6 (> 7.8 pg/mL vs. ≤ 7.8 pg/mL)	2.527 (1.468–4.350)	0.001	2.235 (1.176–4.246)	0.014

## Data Availability

The datasets used and/or analyzed in the current study are available from the corresponding authors on reasonable request.

## References

[1] Sung H., Ferlay J., Siegel R. L (2021). Global Cancer Statistics 2020: GLOBOCAN Estimates of Incidence and Mortality Worldwide for 36 Cancers in 185 Countries. *A Cancer Journal for Clinicians*.

[2] Chen W., Zheng R., Baade P. D (2016). Cancer Statistics in China, 2015. *A Cancer Journal for Clinicians*.

[3] Zhou M., Wang H., Zeng X (2019). Mortality, Morbidity, and Risk Factors in China and its Provinces, 1990-2017: A Systematic Analysis for the Global Burden of Disease Study 2017. *The Lancet*.

[4] Zhou J., Sun H., Wang Z (2020). Guidelines for the Diagnosis and Treatment of Hepatocellular Carcinoma (2019 Edition). *Liver Cancer*.

[5] Chen Y. X., Zhuang Y., Yang P (2020). Helical IMRT-Based Stereotactic Body Radiation Therapy Using an Abdominal Compression Technique and Modified Fractionation Regimen for Small Hepatocellular Carcinoma. *Technology in Cancer Research and Treatment*.

[6] Kim N., Cheng J., Jung I (2020). Stereotactic Body Radiation Therapy vs. Radiofrequency Ablation in Asian Patients With Hepatocellular Carcinoma. *Journal of Hepatology*.

[7] Hinshaw D. C., Shevde L. A. (2019). The Tumor Microenvironment Innately Modulates Cancer Progression. *Cancer Research*.

[8] Kumari N., Dwarakanath B. S., Das A., Bhatt A. N. (2016). Role of Interleukin-6 in Cancer Progression and Therapeutic Resistance. *Tumor Biology*.

[9] Cha H., Lee E. J., Seong J. (2017). Multi-Analyte Analysis of Cytokines That Predict Outcomes in Patients With Hepatocellular Carcinoma Treated With Radiotherapy. *World Journal of Gastroenterology*.

[10] Wei X., Jiang Y., Zhang X (2019). Neoadjuvant Three-Dimensional Conformal Radiotherapy for Resectable Hepatocellular Carcinoma With Portal Vein Tumor Thrombus: A Randomized, Open-Label, Multicenter Controlled Study. *Journal of Clinical Oncology*.

[11] Hu Y., Zhou Y. K., Chen Y. X., Shi S. M., Zeng Z. C. (2017). Clinical Benefits of New Immobilization System for Hypofractionated Radiotherapy of Intrahepatic Hepatocellular Carcinoma by Helical Tomotherapy. *Medical Dosimetry*.

[12] Zeng Z. C., Seong J., Yoon S. M (2017). Consensus on Stereotactic Body Radiation Therapy for Small-Sized Hepatocellular Carcinoma at the 7th Asia-Pacific Primary Liver Cancer Expert Meeting. *Liver Cancer*.

[13] Jiang T., Zeng Z. C., Yang P., Hu Y. (2017). Exploration of Superior Modality: Safety and Efficacy of Hypofractioned Image-Guided Intensity Modulated Radiation Therapy in Patients With Unresectable but Confined Intrahepatic Hepatocellular Carcinoma. *Canadian Journal of Gastroenterology and Hepatology*.

[14] Jang J. W., Oh B. S., Kwon J. H (2012). Serum Interleukin-6 and C-Reactive Protein as a Prognostic Indicator in Hepatocellular Carcinoma. *Cytokine*.

[15] Xu J., Lin H., Wu G., Zhu M., Li M. (2021). IL-6/STAT3 Is a Promising Therapeutic Target for Hepatocellular Carcinoma. *Frontiers in Oncology*.

[16] Wan S., Zhao E., Kryczek I (2014). Tumor-Associated Macrophages Produce Interleukin 6 and Signal Via STAT3 to Promote Expansion of Human Hepatocellular Carcinoma Stem Cells. *Gastroenterology*.

[17] Porta C., De Amici M., Quaglini S (2008). Circulating Interleukin-6 as a Tumor Marker for Hepatocellular Carcinoma. *Annals of Oncology*.

[18] Ozkan H., Yakut M., Karakaya M. F., Erdal H. (2018). Diagnostic and Prognostic Role of Serum Interleukin-6 in Malignant Transformation of Liver Cirrhosis. *Euroasian Journal of Hepato-Gastroenterology*.

[19] Galle P. R., Foerster F., Kudo M (2019). Biology and Significance of Alpha-Fetoprotein in Hepatocellular Carcinoma. *Liver International*.

[20] Loosen S. H., Schulze-Hagen M., Leyh C (2018). IL-6 and IL-8 Serum Levels Predict Tumor Response and Overall Survival After TACE for Primary and Secondary Hepatic Malignancies. *International Journal of Molecular Sciences*.

[21] Gosain R., Anwar S., Miller A., Iyer R., Mukherjee S. (2019). Interleukin-6 as a Biomarker in Patients With Hepatobiliary Cancers. *Journal of Gastrointestinal Oncology*.

[22] Shao Y. Y., Lin H., Li Y. S (2017). High Plasma Interleukin-6 Levels Associated With Poor Prognosis of Patients With Advanced Hepatocellular Carcinoma. *Japanese Journal of Clinical Oncology*.

[23] Myojin Y., Kodama T., Sakamori R (2022). Interleukin-6 Is a Circulating Prognostic Biomarker for Hepatocellular Carcinoma Patients Treated With Combined Immunotherapy. *Cancers*.

[24] Wu Y., Fan W., Xue M (2019). Postintervention Interleukin-6 (IL-6) Level, Rather Than the Pretreatment or Dynamic Changes of IL-6, as an Early Practical Marker of Tumor Response in Hepatocellular Carcinoma Treated With Transarterial Chemoembolization. *The Oncologist*.

[25] Chen Y., Rubin P., Williams J., Hernady E., Smudzin T., Okunieff P. (2001). Circulating IL-6 as a Predictor of Radiation Pneumonitis. *International Journal of Radiation Oncology, Biology, Physics*.

[26] Fu Z. Z., Peng Y., Cao L. Y., Chen Y. S., Li K., Fu B. H. (2016). Correlations Between Serum IL-6 Levels and Radiation Pneumonitis in Lung Cancer Patients: A Meta-Analysis. *Journal of Clinical Laboratory Analysis*.

[27] Matsuoka Y., Nakayama H., Yoshida R (2016). IL-6 Controls Resistance to Radiation by Suppressing Oxidative Stress via the Nrf2-Antioxidant Pathway in Oral Squamous Cell Carcinoma. *British Journal of Cancer*.

[28] Cheng C. C., Ho A. S., Peng C. L (2022). Sorafenib Suppresses Radioresistance and Synergizes Radiotherapy-Mediated Cd8+ T Cell Activation to Eradicate Hepatocellular Carcinoma. *International Immunopharmacology*.

[29] Centurione L., Aiello F. B. (2016). DNA Repair and Cytokines: TGF-β, IL-6, and Thrombopoietin as Different Biomarkers of Radioresistance. *Frontiers in Oncology*.

[30] Chen X., Chen F., Ren Y (2019). IL-6 Signaling Contributes to Radioresistance of Prostate Cancer Through Key DNA Repair-Associated Molecules ATM, ATR, and BRCA 1/2. *Journal of Cancer Research and Clinical Oncology*.

[31] Jarosz-Biej M., Smolarczyk R., Cichoń T., Kułach N. (2019). Tumor Microenvironment as a Game Changer in Cancer Radiotherapy. *International Journal of Molecular Sciences*.

[32] Wu C. T., Chen M. F., Chen W. C., Hsieh C. C. (2013). The Role of IL-6 in the Radiation Response of Prostate Cancer. *Radiation Oncology*.

[33] Chen M. F., Hsieh C. C., Chen W. C., Lai C. H. (2012). Role of Interleukin-6 in the Radiation Response of Liver Tumors. *International Journal of Radiation Oncology, Biology, Physics*.

